# An Optimized *Ustilago maydis* for Itaconic Acid Production at Maximal Theoretical Yield

**DOI:** 10.3390/jof7010020

**Published:** 2020-12-31

**Authors:** Johanna Becker, Hamed Hosseinpour Tehrani, Philipp Ernst, Lars Mathias Blank, Nick Wierckx

**Affiliations:** 1 iAMB—Institute of Applied Microbiology, ABBt—Aachen Biology and Biotechnology, RWTH Aachen University, Worringerweg 1, 52074 Aachen, Germany; johanna.becker@rwth-aachen.de (J.B.); hamed.tehrani@rwth-aachen.de (H.H.T.); lars.blank@rwth-aachen.de (L.M.B.); 2 Institute of Bio- and Geosciences IBG-1: Biotechnology, Forschungszentrum Jülich, 52425 Jülich, Germany; p.ernst@fz-juelich.de

**Keywords:** itaconic acid, *U. maydis*, metabolic engineering, fungi, yeast

## Abstract

*Ustilago maydis*, a member of the Ustilaginaceae family, is a promising host for the production of several metabolites including itaconic acid. This dicarboxylate has great potential as a bio-based building block in the polymer industry, and is of special interest for pharmaceutical applications. Several itaconate overproducing *Ustilago* strains have been generated by metabolic and morphology engineering. This yielded stabilized unicellular morphology through *fuz7* deletion, reduction of by-product formation through deletion of genes responsible for itaconate oxidation and (glyco)lipid production, and the overexpression of the regulator of the itaconate cluster *ria1* and the mitochondrial tricarboxylate transporter encoded by *mttA* from *Aspergillus* *terreus*. In this study, itaconate production was further optimized by consolidating these different optimizations into one strain. The combined modifications resulted in itaconic acid production at theoretical maximal yield, which was achieved under biotechnologically relevant fed-batch fermentations with continuous feed.

## 1. Introduction

Itaconic acid and its derivatives are found in many application fields, such as the production of paper, paints, and fibers, or in waste water treatment [1,2,3,4] providing a stable market for this bio-based chemical. In 2004, the organic acid was classified as one of the top 12 value-added platform chemicals derived from biomass [5]. There is also a strong interest in this molecule in the medical and pharmaceutical sectors, both as an antibacterial compound [6] and as an immunoregulator for the treatment of autoimmune diseases [7] and viral infections including SARS-COV2 [8]. However, its relatively high production cost compared to fossil counterparts like acrylic acid prevents an even more expanded usage range [9,10]. Given the already high yield, titer, and rate of the industrially established itaconic acid production process with *Aspergillus terreus* [11], a qualitative breakthrough in other dimensions of the process window is needed. Non-conventional itaconate-producing yeasts like *Ustilago*, *Candida*, or *Pseudozyma* [12,13,14] may offer such a breakthrough because of their unicellular morphology as well as their lower sensitivity to medium impurities. It also enables easier handling and scale-up using cheaper raw substrates or even waste streams [10,15,16]. However, compared to the filamentous ascomycete *A. terreus*, the product to substrate yield has to be improved to make *U. maydis* competitive, and this yield also needs to be achievable under industrially relevant conditions [17].

In nature, *U*. *maydis* is known for its pathogenicity towards maize (*Zea mays*) causing corn smut disease [18,19]. To penetrate and invade maize tissue, *U. maydis* switches from a yeast-like, non-pathogenic to a filamentous, pathogenic cell form [20]. This switch is governed by a complex regulatory pathway which has been investigated in detail (see [21,22] for extensive reviews). Although predominantly growing in its yeast form in fermentation processes, *U. maydis* can switch to filamentous growth under stress conditions such as the presence of hydrophobic lipids, low pH, or nitrogen deficiency [23,24,25]. Filamentous growth causes issues such as high viscosity, reduced oxygen supply, and cell adherence to reactor walls [9]. This can be avoided through the deletion of *fuz7*, the product of which plays an essential role in regulating pathogenicity and the switch to filamentous growth [23]. This deletion stabilizes the yeast-like morphology of *U. maydis* and *U. cynodontis* without impacting the fitness of the cells under biotechnologically relevant stresses [26,27].

*U*. *maydis* produces itaconate as one product from a potpourri of metabolites including organic acids such as malate, succinate, and (S)-2-hydroxyparaconate, polyols such as erythritol and mannitol, and different lipidic products including glycolipids and triglycerides [28,29,30,31,32,33]. It can also metabolize a range of renewable carbon sources, which besides sugar also include glycerol [34], galacturonic acid [35], cellulose [36], xylan [37], and pectin [38]. Although these features make *U. maydis* an attractive candidate for industrial applications [9,39,40], it also poses a drawback because often multiple products are produced simultaneously. This hinders handling and downstream processing, and it also reduces yield on substrate by diverting carbon flux away from the main product [41]. If itaconic acid is to become a bulk chemical, yield is the most relevant production parameter because substrate cost is the decisive price-determining factor. For itaconate production from glucose, the reported maximal theoretical yield is 0.72 g_ITA_ g_glu_^−1^, which equals 1 mol_ITA_ mol _glu_^−1^. The yield achieved in practice is affected by different factors such as cell density, the metabolic pathway leading up to itaconate (i.e., anaplerosis), bottlenecks in the itaconate biosynthesis pathway itself, side product formation, redox cofactor balancing, and cell maintenance demand [42,43].

In previous studies several knockouts, promotor replacements and overexpression of genes were implemented to increase itaconate production, reduce by-product formation, and stabilize the unicellular morphology [10,26,41,44,45]. Those metabolic engineering approaches resulted in several itaconate hyper-producing *Ustilago* strains with individual modifications. In this study, these modifications are consolidated into one strain, based on the previously engineered *U. maydis* ITA chassis [41]. The resulting strain K14 produces itaconate from glucose at maximum theoretical yield. The catalytic vigor of the strain was demonstrated in fed-batch cultures.

## 2. Materials and Methods

### 2.1. Media and Culture Conditions

All strains used in this work are listed in Table 1. *U. maydis* strains were grown in YEPS medium containing 10 g L^−1^ yeast extract, 10 g L^−1^ peptone, and 10 g L^−1^ sucrose. As screening medium for production experiments, *U. maydis* was cultivated in modified Tabuchi medium (MTM) according to Geiser et al. [30]. Besides varying glucose and buffer (2-(N-morpholino) ethanesulfonic acid (MES; pH adjusted to 6.5 with NaOH) or CaCO_3_) concentrations, this medium contained 0.8 g L^−1^ NH_4_Cl, 0.2 g L^−1^ MgSO_4_·7H_2_O, 0.01 g L^−1^ FeSO_4_·7H_2_O, 0.5 g L^−1^ KH_2_PO_4_, 1 mL L^−1^ vitamin solution, and 1 mL L^−1^ trace element solution. The vitamin solution contained (per liter) 0.05 g D-biotin, 1 g D-calcium panthotenate, 1 g nicotinic acid, 25 g myo-inositol, 1 g thiamine hydrochloride, 1 g pyridoxol hydrochloride, and 0.2 g para-aminobenzoic acid. The trace element solution contained (per liter) 1.5 g EDTA, 0.45 g ZnSO_4_·7H_2_O, 0.10 g MnCl_2_·4H_2_O, 0.03 g CoCl_2_·6H_2_O, 0.03 g CuSO_4_·5H_2_O, 0.04 g Na_2_MoO_4_·2H_2_O, 0.45 g CaCl_2_·2H_2_O, 0.3 g FeSO_4_·7H_2_O, 0.10 g H_3_BO_3_, and 0.01 g KI. When using CaCO_3,_ medium components were added relative to the total volume of solids plus liquid, leading to a higher aqueous concentration of soluble components. Shaking cultures of *U. maydis* were performed in 24-well System Duetz^®^ plates with a filling volume of 1.5 mL (shaking diameter = 50 mm, *n* = 300 rpm, T = 30 °C and Φ = 80%) [46] or in 500 mL shaking flasks with a filling volume of 50 mL (shaking diameter = 25 mm, *n* = 200 rpm, T = 30 °C and Φ = 80%). When using System Duetz^®^, cultures were inoculated in parallel into multiple plates in order to ensure continuous oxygenation by taking a complete plate as sacrificial sample for each sample point.

Pulsed fed-batch fermentations were performed in New Brunswick BioFlo^®^ 115 bioreactors (Eppendorf, Germany) as described in [10]. Fed-batch fermentations with continuous feed were performed in a 2.0 L DASGIP^®^ Bioblock bioreactor (Eppendorf, Germany) with a starting volume of 1.0 L. The medium contained 120 g L^−1^ glucose, 0.8 g L^−1^ or 4 g L^−1^ NH_4_Cl, 0.2 g L^−1^ MgSO_4_·7H_2_O, 0.01 g L^−1^ FeSO_4_·7H_2_O, 0.5 g L^−1^ KH_2_PO_4_, 1 mL L^−1^ vitamin solution as specified above, 1 mL L^−1^ trace element solution as specified above, and 1 g L^−1^ yeast extract. When the glucose concentration reached approximately 50 g L^−1^, a constant feed of a 50% glucose solution was started. Feeding rates of 0.75 and 2.8 g h^−1^ were estimated from the glucose consumption rates of previous pulsed fed-batch fermentations under similar conditions. During cultivation, the pH was controlled by automatic addition of 5 M NaOH and 1 M HCl. 0.5 mL Antifoam 204 (Sigma Life Science, St. Louis, O, USA) was added manually every 24 h to avoid foam formation. The dissolved oxygen (DO) was controlled at 30% by using a cascade mode including stirring 800–1200 rpm, air flow 1-2 L min^−1^ and the addition of pure oxygen. The CO_2_ formation was determined with the DASGIP GA4 module, employing infrared (IR) sensors (BlueSens). The cultivations were performed at 30 °C. Bioreactors were inoculated to an optical density measured at a wavelength of 600 nm (OD_600_) of 0.75 from a 48 h preculture in 50 mL MTM.

### 2.2. Analytical Methods

When using CaCO_3_ as buffer, 1 mL culture broth was taken for OD_600_ and high-performance liquid chromatography (HPLC) analysis. The CaCO_3_ was dissolved 1:1 with 4 M HCl prior to further measurements as described in Zambanini et al. [49]. Cell densities were measured by determining the absorption at 600 nm with an Ultrospec 10 Cell Density Meter (Amersham Biosciences, UK).

For HPLC analysis, all samples were filtered with Rotilabo^®^ (CA, 0.2 µm, Ø 15 mm) or Acrodisc^®^ (GHP, 0.2 µm, Ø 13 mm) syringe filters and diluted 1:5 or 1:10 with 5 mM H_2_SO_4_ or deionized water. Products in the supernatant were analyzed using a DIONEX UltiMate 3000 HPLC System (Thermo Scientific, Waltham, Massachusetts, USA) or a Agilent 1260 Infinity HPLC system (Agilent, Waldbronn, Germany) with an ISERA Metab-AAC column 300 × 7.8 mm column (ISERA, Düren, Germany). As mobile phase, 5 mM H_2_SO_4_ with a constant flow rate of 0.6 mL min^−1^ and a temperature of 40 °C was used. When using the DIONEX UltiMate 3000 HPLC System, detection was carried out by a DIONEX UltiMate 3000 Variable Wavelength Detector set to 210 nm and a SHODEX RI-101 detector (Showa Denko Europe GmbH, Munich, Germany). When using the Agilent 1260 Infinity HPLC system, detection was undertaken by a diode array detector (DAD) at 210 nm and a refraction index (RI) detector. Analytes were identified via retention time and ultraviolet (UV)/RI ratio compared to corresponding standards.

All values are the arithmetic mean of at least two biological replicates. For *n* = 2, error bars indicate the deviation from the mean and for n > 2 error bars indicate the standard error of the mean. Statistical significance was evaluated by t test (two-tailed distribution, heteroscedastic, *p* ≤ 0.05).

### 2.3. Plasmid Cloning and Strain Engineering

Plasmids were constructed by Gibson assembly [50] using the NEBuilder^®^ HiFi DNA Assembly Cloning Kit (New England Biolabs (NEB), Ipswich, MA, USA). Primers were ordered as DNA oligonucleotides from Eurofins Genomics (Ebersberg, Germany). As polymerase, Q5^®^ High-Fidelity DNA Polymerase (NEB) was used. Detailed information about utilized primers and plasmids are listed in Table 2 and Supplemental Table S1. Competent *E. coli* DH5α were used for standard cloning and plasmid maintenance according to Sambrook et al. [51]. Plasmids were confirmed by polymerase chain reaction (PCR) or sequencing. Generation of protoplasts and transformation of *U. maydis* were performed according to Brachmann et al. [52]. Genomic DNA of *U. maydis* was isolated according to Hoffman et al. [53]. For the deletion of *fuz7*, homologous recombination with 1000 bp flanking regions including FRT-sites and a hygromycin resistance cassette were used [54]. Successful integration and deletion was verified by PCR and sequencing. For the overexpression of *mttA*, the plasmid *P_etef_*-Cbx-*AT_mttA* was used [48].

Quantitative PCR was applied to determine the copy number of *mttA* integrated into the *U. maydis* genome using Luna^®^ Universal qPCR Master Mix (NEB, Frankfurt, Germany). Primers were designed using “GenScript Real-Time PCR (TaqMan) Primer Design” tool (Gen Script, Piscataway, New Jersey, USA). Primer sequences are given in Table 3. As reference genes, UMAG_02592 and UMAG_03726 were amplified with the primer pairs JB-126/JB-127 and JB-128/JB-129. Primers JB-132/JB-133 specifically bound within the *mttA* sequence (Supplemental Table S1). Amplification curves were taken by Bio-Rad CFX ConnectTM Real-Time PCR Detection system and data were analyzed by using Bio-Rad CFX ManagerTM 3.1 software (Bio-Rad Laboratories, Hercules, CA, USA) using the ∆Ct method according to Pfaffl [55].

## 3. Results and Discussion

### 3.1. Prevention of Filamentous Growth by fuz7 Deletion and Its Influence on Itaconate Production

**Figure 1:**
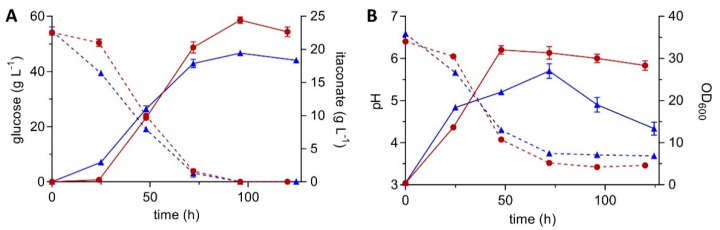
System Duetz^®^ cultivation of morphology-engineered *U. maydis* MB215 strains in modified Tabuchi medium (MTM) with 50 g L^−1^ glucose and 100 mM (2-(N-morpholino) ethanesulfonic acid (MES), incubated in 24-well plates with a filling volume of 1.5 mL (shaking diameter = 50 mm, *n* = 300 rpm, T = 30 °C and Φ = 80%). (**A**) Concentrations of itaconate (continuous lines) and glucose (dotted lines), (**B**) optical density measured at a wavelength of 600 nm (OD_600,_ continuous lines) and pH (dotted lines) of *U. maydis* MB215 ∆*cyp3* ∆MEL ∆UA ∆*dgat* ∆*P_ria1_*::*P_etef_* (▲) and the same strain with additional *fuz7* deletion (●). Error bars indicate the standard error of the mean (*n* = 3).

In previous work, itaconate production with *Ustilago* has been significantly improved. The characterization and upregulation of the itaconate gene cluster [44,56,57] as well as the engineering of the mitochondrial carrier for *cis-*aconitate [48] has laid the foundation for this improvement. Those achievements were combined with further modifications including the deletion of genes responsible for itaconate oxidation (*cyp3*) and (glyco)lipid production (MEL, UA, *dgat*), resulting in the *U. maydis* MB215 ITA chassis (∆*cyp3* ∆MEL ∆UA ∆*dgat* ∆*P_ria1_*::*P_etef_*) with reduced by-product formation [41]. Filamentous growth was observed for this strain, similar to other engineered *U. maydis* variants [10]. The morphology switch is likely induced by the additional stress imposed by the metabolic engineering itself, and the resulting high itaconate titers and associated low pH. This drawback can be overcome by the deletion of *fuz7* (UMAG_01514) [26,27]. The influence of this knockout in the *U. maydis* MB215 ITA chassis was assessed in System Duetz^®^ cultivations in MTM containing 50 g L^−1^ glucose and 100 mM MES (Figure 1). Under these conditions, the ∆*fuz7* strain produced 1.3-fold more itaconate than the reference ITA chassis strain (Figure 1A, Table 3). Full consumption of glucose by both strains resulted in an equivalently improved yield of the ∆*fuz7* strain to 0.45 ± 0.01 g_ITA_ g_glu_^−1^. The overall production rate was increased by 12% and the maximal rate even by 26% (Table 3).

These results clearly illustrate the benefit of preventing filamentous growth. In these deeply engineered strains of *U. maydis*, cells start to adhere to the walls of the culture plates during the production phase, likely as a result of the combined stress of ammonium limitation, low pH and increasing product concentrations [23,24,25]. This adhesion has been described in detail in Hosseinpour-Tehrani et al. [10]. The resulting cell accumulations likely encounter oxygen, buffer, and nutrition heterogeneities, leading to limitations for cells deeper within the clumps. The accumulation on the walls was reflected in decreasing optical density values after 72 h (Figure 1B) and a decreasing itaconate productivity (Figure 1A). This is in contrast to the *fuz7* mutant, which showed a constant itaconate production rate in the production phase until glucose was depleted.

### 3.2. Overexpression of mttA from A. terreus and Its Impact on Itaconate Production

The transport of *cis*-aconitate from the mitochondria to the cytosol is the rate-limiting step in the itaconate production pathway of *U. maydis* [44,56]. In the ITA chassis, this bottleneck is addressed through the overexpression of *mtt1* caused by the promoter exchange of the itaconate cluster regulator encoded by *ria1* [41,56]. However, the *mttA* transporter from *A. terreus* was shown to cause a higher metabolic flux towards itaconate than its *U. maydis* counterpart *Mtt1* [10,48]. Overexpression of both *mtt1* and *mttA* further enhances itaconate production. Therefore, a *P_etef_mttA* construct was targeted to the *ip*-locus on the genome of the novel ∆*fuz7* strain.

**Figure 2:**
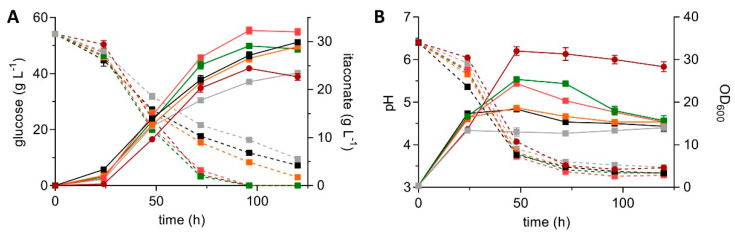
System Duetz^®^ cultivation of *U. maydis* MB215 strains expressing *mttA* in MTM with 50 g L^−1^ glucose and 100 mM MES, incubated in 24-well plates with a filling volume of 1.5 mL (shaking diameter = 50 mm, *n* = 300 rpm, T = 30 °C and Φ = 80%). Itaconate production (continuous lines) and glucose consumption (dotted lines) (**A**) are shown, as well as OD_600_ (continuous lines) and pH (dotted lines) (**B**) of *U. maydis* MB215 ∆*cyp3* ∆MEL ∆UA ∆*dgat* ∆*P_ria1_*::*P_etef_* ∆*fuz7* (red) and five P_etef_*mttA* transformants named K3 (grey), K8 (green), K9 (orange), K10 (pink), and K14 (black). Error bars indicate the standard error of the mean (*n* = 3).

Targeted integration of CbxR constructs into the *ip*-locus is not perfect. Often, multicopy integration and/or ectopic insertion into random genomic sites occurs. Therefore, several clones were picked to identify the best itaconate producer. Insertion of at least one *mttA* copy was verified by PCR for five clones resulting in the strains listed in Table 3. The strains significantly differed from each other regarding itaconate production, glucose consumption, and growth (Figure 2).

With the exception of K3, all *mttA* transformants outperformed the reference strain with 18–32% improvements in titer (Figure 2A, Table 3). Clones K3, K9, and K14 did not consume all glucose during 120 h of cultivation (Figure 2A), which further boosted the yield. All *mttA* transformants achieved a higher yield than the reference (Table 3), with K14 reaching 0.64 ± 0.03 g_ITA_ g_Glu_^−1^, which is 89% of the theoretical maximum. Strains K8 and K10 had the highest rates, which were 25% and 32% higher, respectively, compared to the reference. Overexpression of *mttA* also had a strong impact on the growth (Figure 2B). While *U. maydis* MB215 ∆*cyp3* ∆MEL ∆UA ∆*dgat* ∆*P_ria1_*::*P_etef_* ∆*fuz7* reached OD_600_ values above 30, all *mttA* transformants remained well below this value. A similar effect was observed in other *mttA* overexpressing strains, where growth and glucose consumption were also strongly decreased [48]. The constitutive P*_etef_* promoter causes expression of m*ttA* during the growth phase [58]. It is assumed that this forces *cis*-aconitate export from the mitochondria to the cytosol, leading to the observed growth defects.

**Figure 3:**
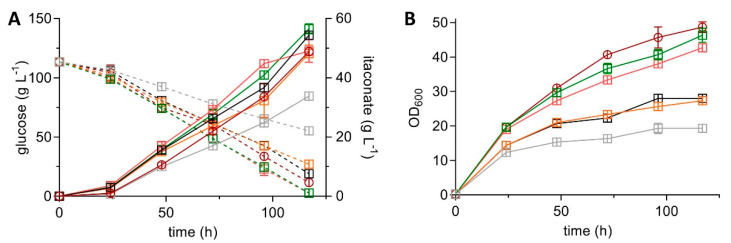
System Duetz^®^ cultivation of six *U. maydis* MB215 mutants in MTM with 100 g L^−1^ glucose and 66 g L^−1^ CaCO_3_, incubated in 24-well plates with a filling volume of 1.5 mL (shaking diameter = 50 mm, *n* = 300 rpm, T = 30 °C and Φ = 80%). Concentrations of itaconate (continuous lines) and glucose (dotted lines) (**A**) are shown, as well as OD_600_ (**B**) of *U. maydis* MB215 ∆*cyp3* ∆MEL ∆UA ∆*dgat* ∆*P_ria1_*::*P_etef_* ∆*fuz7* (red) and five P_etef_*mttA* transformants *U. maydis* MB215 ∆*cyp3* ∆MEL ∆UA ∆*dgat* ∆*P_ria1_*::*P_etef_* ∆*fuz7 P_etef_mttA*, K3 (grey), K8 (green), K9 (orange), K10 (pink), and K14 (black). Error bars indicate the standard error of the mean (*n* = 3).

The trends regarding OD_600_, yield, and rate were similar under screening conditions with a higher substrate concentration and CaCO_3_ as buffer (Figure 3, Table 3). Much higher titers of up to 56.5 ± 1.7 g L^−1^ were reached compared to the MES-buffered cultivation due to the higher buffer capacity of CaCO_3_, higher substrate concentration, and in situ precipitation of calcium itaconate, which alleviates product inhibition.

### 3.3. Correlation between Copy Number of P_etef_mttA and Impact on Itaconate Production

In order to test whether these differences in production parameters and growth are a result of differences in the copy number of *P_etef_mttA*, the copy number was determined by quantitative PCR. Primer efficiencies and C_t_ values of *mttA* and two reference genes, UMAG_02595 and UMAG_03726, were 2.014 for JB-126/JB-127 (R^2^ = 0.999), 1.994 for JB-128/JB-129 (R^2^ = 0.999), and 1.976 for JB-132/JB-133 (R^2^ = 1.0). Ratios between *mttA* and each reference gene were calculated independently according to Pfaffl et al. [55] and the resulting mean of both ratios was rounded to an integer value. As positive control, *U. maydis* MB215 ∆UMAG_05079 *P_etef_mttA* was used. This strain was previously proven to be a single-copy *mttA* transformant by Southern blot [48], which was confirmed by the qPCR method. For the *mttA* transformants, copy numbers between 1 and 4 were determined (Table 4).

**Figure 4:**
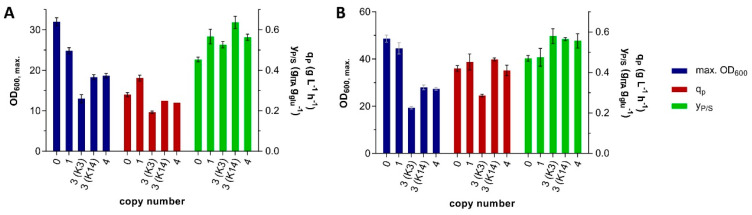
Production parameters of *U. maydis* MB215 ∆*cyp3* ∆MEL ∆UA ∆*dgat* ∆*P_ria1_*::*P_etef_* ∆*fuz7* transformants with different *mttA* copy numbers incubated in MTM with 50 g L^−1^ glucose and 100 mM MES (**A**) and with 100 g L^−1^ glucose and 66 g L^−1^ CaCO_3_ (**B**). Error bars indicate the standard error of the mean (*n* = 3).

Cells of strain K8 with a single *mttA* copy reached the highest OD_600_ and itaconate production rate (Figure 4). The transformants with higher copy numbers showed lower OD_600_ and production rates, but significantly higher yields. Transformant K3 represents an outlier, which is most apparent in the direct comparison to K14 having the same copy number of 3. Possibly, one or more copies of the *mttA* construct were inserted into a different locus, which may lead to different expression levels or to defects in growth due to gene disruption. Overall, transformant K14 showed the best balance of high yield with minimal reduction in growth and production rates. Therefore, this strain was selected for further characterization.

### 3.4. Evaluation of Itaconate Production with the Novel Engineered Strain K14 in a Bioreactor

The final consolidated *U. maydis* MB215 ∆*cyp3* ∆MEL ∆UA ∆*dgat* ∆*P_ria1_*::*P_etef_* ∆*fuz7 P_etef_mttA*_K14, henceforth named strain K14 for ease of reference, was deeply engineered to reduce by-product formation, to stabilize the yeast-like morphology, and to alleviate bottlenecks in the itaconate production pathway. This engineering strongly impacted growth, and thus might also affect its catalytic vigor under stress. To assess the performance of the novel engineered strain K14 under more industrially relevant conditions, fed-batch bioreactor experiments were performed.

**Figure 5:**
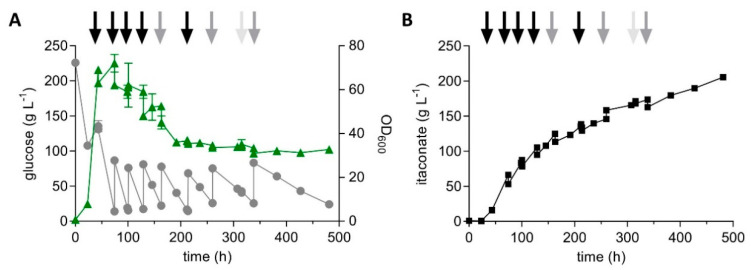
High-density pulsed fed-batch fermentation of *U. maydis* strain K14. (A) concentration of glucose (●) and OD_600_ values (▲) and (B) concentration of itaconate (■) during fermentation in a bioreactor containing batch medium (200 g L^−1^ glucose, 4 g L^−1^ NH_4_Cl, CaCO_3_ as buffering agent, 30 °C, 1000 rpm, top pitched blade impeller, bottom Rushton impeller). Arrows indicate the addition of 80 g glucose + 50 g CaCO_3_ (black arrow), 80 g glucose (grey arrow), or 50 g CaCO_3_ (light grey arrow). Error bars indicate the deviation from the mean (*n* = 2).

Previously, in situ crystallization of calcium itaconate was successfully used to achieve very high titers with *U. maydis* MB215 ∆*cyp3* ∆*P_ria1_*::*P_etef_* ∆*fuz7 P_etef_mttA* [10]. Under similar conditions with 200 g L^−1^ glucose and 4 g L^−1^ NH_4_Cl in the presence of CaCO_3_, strain K14 achieved very similar yield, titer and rate of itaconate production (Supplemental Table S2). A titer of 205.6 ± 1.1 g L^−1^ itaconate was achieved within 481 h with an overall productivity of 0.43 ± 0.00 g L^−1^ h^−1^ and a yield of 0.32 ± 0.00 g_ITA_ g_glu_^−1^ (Figure 5). Although a very high titer was reached under these conditions due to the alleviation of product inhibition, the yield is relatively low compared to the shaken cultures. After the fermentation, extensive clumping of solids attached to reactor walls and components was discovered, indicating that the shown itaconate production from broth samples during cultivation may be an underestimation. Although the in situ precipitation of itaconate is a very promising strategy, it clearly requires extensive optimization of solids feeding and reactor mixing in order to realize the full potential of the engineered strain.

**Figure 6:**
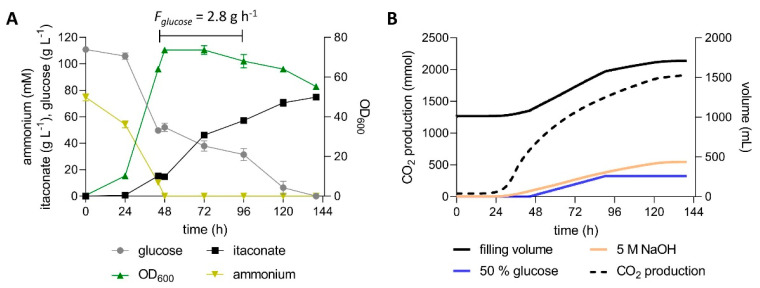
High-density fed-batch fermentation with continuous feed of *U. maydis* strain K14. (**A**) concentration of glucose, itaconate and ammonium, and OD_600_ values and (**B**) filling volume, CO_2_ production and the added volumes of 5 M NaOH and 50% glucose during fermentation in a bioreactor containing batch medium with 120 g L^−1^ glucose and 4 g L^−1^ NH_4_Cl. The pH was kept at 6.5 by automatic titration with NaOH. Cultures were fed with an additional 130 g glucose at a rate of 2.8 g h^−1^ during the indicated time interval. Error bars indicate the deviation from the mean (*n* = 2).

**Figure 7:**
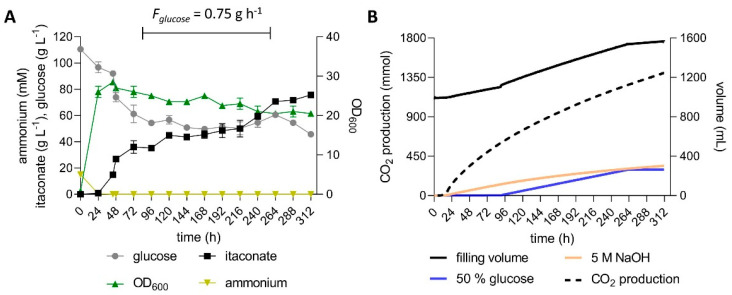
Low-density fed-batch fermentation with continuous feed of *U. maydis* strain K14. (**A**) concentration of glucose, itaconate and ammonium, and OD_600_ values and (**B**) filling volume, CO_2_ production and the added volumes of 5 M NaOH and 50% glucose during fermentation in a bioreactor containing batch medium with 120 g L^−1^ glucose, 0.8 g L^−1^ NH_4_Cl. The pH was kept at 6.5 by automatic titration with NaOH. Cultures were fed with an additional 130 g glucose at a rate of 0.75 g h^−1^ during the indicated time interval. Error bars indicate the deviation from the mean (*n* = 2).

The intermittent stress imposed by the pulsed feeding strategy likely also negatively affected production. This is especially apparent in NaOH-titrated pulsed fed-batch fermentations, where only 60 g L^−1^ itaconate was produced with a yield of 0.42 g_ITA_ g_glu_^−1^ (Supplemental Figure S1). Although this is a considerable improvement compared to a similar fermentation with *U. maydis* MB215 ∆*cyp* ∆*P_ria1_*::*P_etef_* ∆*fuz7 P_etef_mttA* [10] (Supplemental Table S2), the achieved yield is still far lower than that obtained in shake flasks. This indicates that the strain modifications, especially the overexpression of *mttA*, reduced the tolerance of the engineered strains to osmotic stress. Consequently, the novel engineered strain was cultivated in fed-batch fermentations with continuous feed to achieve a lower baseline glucose concentration. High-density fermentations with 4 g L^−1^ NH_4_Cl (Figure 6) as well as low-density fermentations with 0.8 g L^−1^ NH_4_Cl (Figure 7) were performed. A starting glucose concentration of 120 g L^−1^ was allowed to drop to approximately 50 g L^−1^ during growth, at which point a constant feed of 0.75 or 2.8 g h^−1^ was started, for the low and high-density fermentation, respectively.

In the high density fermentation, 74.9 ± 1.25 g L^−1^ itaconate was produced by the *U. maydis* strain K14 within 140 h with an overall productivity of 0.53 ± 0.01 g L^−1^ h^−1^ and a yield of 0.54 ± 0.02 g_ITA_ g_glu_^−1^ (Figure 6). The low density fermentation resulted in a similar itaconate titer of 75.7 ± 1.3 g L^−1^. The five-fold reduction in NH_4_Cl as growth limiting nutrient only resulted in an approximately two-fold reduction of the maximum OD_600_ value as well as of the overall production rate (Supplemental Table S2). A similar trend was observed previously for both itaconate- and malate-producing strains [44,49]. The lower substrate requirement for biomass formation enabled a higher yield of 0.66 ± 0.02 g_ITA_ g_glu_^−1^ (Figure 7). Compared to the low-density pulsed fed batch (Supplemental Figure S1), the fed batch with continuous feed increased the titer by 27%, the overall productivity by 14% and the yield by 57%. These results clearly illustrate the benefit of the lower glucose concentration, probably due to a reduced osmotic stress and the absence of osmo shocks caused by glucose pulses. A similar trend was observed for an engineered *U. cynodontis* strain, where a constant glucose feed controlled by an in-line glucose sensor significantly increased the production parameters, while lowering the production of erythritol as an osmoprotectant [27]. Erythritol production was not observed in our *U. maydis* cultures, which is in good accordance with previous studies [28,30]. However, this species can tolerate over 2.5 osmol L^−1^ [3] and is known to produce other osmotically active compounds when exposed to such extreme osmotic conditions. Salmerón-Santiago et al. [59] reported that *U. maydis* cells treated with 1 M sorbitol accumulated an increased level of trehalose, probably functioning as an osmoprotectant. The trehalase activity in these cells was increased at the same time, indicating cellular mechanisms for a rapid adaption of the trehalose content. Cervantes-Chávez et al. [60] found that *U. maydis* mutants with a disrupted trehalose biosynthesis pathway were more sensitive to osmotic stress than the wildtype. Assuming that the itaconate produced and the 0.5–1 M glucose pulses had a similar effect, it is reasonable to assume that the *U. maydis* cells were stressed during the pulsed fed-batch fermentations. This stress, combined with the drain of carbon posed by the synthesis of compatible solutes, likely caused the lower itaconate yield.

The yield of 0.66 ± 0.02 g_ITA_ g_glu_^−1^ is the highest yield ever reported for *U. maydis*, and it is also higher than most reported yields achieved with *A. terreus* [43]. In fact, the low-density fermentation achieved the theoretical maximal yield during the production phase. When disregarding the glucose consumed during the first 24 h in the growth phase, the yield was 0.72 ± 0.02 g_ITA_ g_glu_^−1^, or 1.00 ± 0.03 mol_ITA_ mol_glu_^−1^.

## 4. Conclusions

This study explored the limits of microbial itaconic acid production with *U. maydis* by combinatorial metabolic and morphological engineering. These modifications, especially the overexpression of *mttA*, had a major effect on growth of the final strain *U. maydis* K14. This reduction in growth affected its performance in fed-batch reactors, but this effect could be avoided by reducing the glucose concentration with a continuous feeding strategy. Under these conditions, itaconate was produced from glucose at 100% of the theoretical maximum yield during the production phase in a low-density fermentation. Looking forward, osmotolerance of *U. maydis* may be enhanced by laboratory evolution. Furthermore, production of itaconic acid at low pH values is also paramount. Although *U. maydis* grows relatively poorly at low pH, the shaken cultures clearly indicate that the K14 strain is able to produce itaconic acid at pH levels below 4. A pH shift between growth and production may therefore enable low-pH production. Since substrate cost is usually the main price determining factor for commodity products, the high yield achieved in this work will significantly contribute to the establishment of an *Ustilago-*based industrial itaconate production process, further enabled by the facile, yeast-like growth of this strain.

## Figures and Tables

**Table 1:** *U. maydis* MB215 strains used in this study.

Strain Designation	Resistance	Reference
*U. maydis* MB215		[47]
*U. maydis* MB215 ∆*cyp3* ∆*P_ria1_*::*P_etef_*		[10]
*U. maydis* MB215 ∆UMAG_05079 *P_etef_mttA*	hyg^R^, cbx^R^	[48]
*U. maydis* MB215 ∆*cyp3* ∆*fuz7* ∆*P_ria1_*::*P_etef_ P_etef_mttA*_K14	hyg^R^, cbx^R^	[10]
*U. maydis* MB215 ∆*cyp3* ∆MEL ∆UA ∆*dgat* ∆*P_ria1_*::*P_etef_* (=ITA chassis)		[41]
*U. maydis* MB215 ∆*cyp3* ∆MEL ∆UA ∆*dgat* ∆*P_ria1_*::*P_etef_* ∆*fuz7*		this study
*U. maydis* MB215 ∆*cyp3* ∆MEL ∆UA ∆*dgat* ∆*P_ria1_*::*P_etef_* ∆*fuz7 P_etef_mttA*_K3	cbx^R^	this study
*U. maydis* MB215 ∆*cyp3* ∆MEL ∆UA ∆*dgat* ∆*P_ria1_*::*P_etef_* ∆*fuz7 P_etef_mttA*_K8	cbx^R^	this study
*U. maydis* MB215 ∆*cyp3* ∆MEL ∆UA ∆*dgat* ∆*P_ria1_*::*P_etef_* ∆*fuz7 P_etef_mttA*_K9	cbx^R^	this study
*U. maydis* MB215 ∆*cyp3* ∆MEL ∆UA ∆*dgat* ∆*P_ria1_*::*P_etef_* ∆*fuz7 P_etef_mttA*_K10	cbx^R^	this study
*U. maydis* MB215 ∆*cyp3* ∆MEL ∆UA ∆*dgat* ∆*P_ria1_*::*P_etef_* ∆*fuz7 P_etef_mttA*_K14 (=K14 strain)	cbx^R^	this study

**Table 2:** Plasmids used in this study.

Plasmid	Description	Reference
pJET1.2/blunt	Ori ColE1; AmpR	Thermo Scientific, Germany
pFLPexpC	*P_crg1_* promoter; synthetic FLP recombinase gene; CbxR; ARS; AmpR	Prof. M. Feldbrügge, Heinrich-Heine University Düsseldorf, Germany
pUMa1523	FRTm1-HygR-FRTm1 cassette; GentR	Dr. K. Schipper, Heinrich-Heine University Düsseldorf, Germany
pJET1.2-*fuz7* 5′-UTR flank -FRTm1-HygR-FRTm1-*fuz7* 3′-UTR flank	pJET1.2 with 5′- and 3′-UTR flank of UMAG_01514 as deletion construct; HygR; FRT m1 recombination sites	this study
*P_etef_* -Cbx-*AT_mttA*	constitutive *P_etef_* promoter, dicodon-optimized version of *A. terreus* ATEG_09970 (*mttA*), cbxR, ampR	[48]

**Table 3:** ITA production parameters of engineered strains of the *U. maydis* MB215 ITA chassis (∆*cyp3* ∆MEL ∆UA ∆*dgat* ∆*P_ria1_*::*P_etef_*). ±values indicate the standard error of the mean (*n* = 3). Symbols refer to Figure 1, Figure 2 and Figure 3.

Conditions.	Symbol	Strain Modification	ITA Titer_max_ (g L^−1^)	q_P_^a^ (g L^−1^ h^−1^)	q_P,max_^b^ (g L^−1^ h^−1^)	y_P/S_^c^ (g_ITA_ g_glu_^−1^)
100 mM MES, 50 g L^−1^ glucose	▲	control	19.4 ± 0.3	0.25 ± 0.01	0.35 ± 0.02	0.36 ± 0.02
	●	∆*fuz7*	24.4 ± 0.5	0.28 ± 0.01	0.44 ± 0.03	0.45 ± 0.01
	■	*P_etef_mttA*_K3	23.5 ± 0.6	0.20 ± 0.00	0.46 ± 0.02	0.53 ± 0.01
	■	*P_etef_mttA*_K8	29.1 ± 0.1	0.35 ± 0.01	0.49 ± 0.03	0.54 ± 0.01
	■	*P_etef_mttA*_K9	28.9 ± 0.3	0.24 ± 0.00	0.44 ± 0.01	0.57 ± 0.01
	■	*P_etef_mttA*_K10	32.3 ± 0.8	0.37 ± 0.00	0.54 ± 0.01	0.60 ± 0.02
	■	*P_etef_mttA*_K14	29.9 ± 0.7	0.25 ± 0.00	0.44 ± 0.01	0.64 ± 0.03
66 g L^−1^ CaCO_3_, 100 g L^−1^ glucose	**○**	∆*fuz7*	48.8 ± 1.3	0.42 ± 0.01	0.70 ± 0.07	0.47 ± 0.01
	**□**	*P_etef_mttA*_K3	33.8 ± 0.5	0.29 ± 0.00	0.43 ± 0.06	0.58 ± 0.04
	**□**	*P_etef_mttA*_K8	56.5 ± 1.7	0.48 ± 0.01	0.74 ± 0.09	0.51 ± 0.02
	**□**	*P_etef_mttA*_K9	48.1 ± 2.9	0.41 ± 0.02	0.52 ± 0.02	0.56 ± 0.03
	**□**	*P_etef_mttA*_K10	49.0 ± 3.8	0.42 ± 0.03	0.64 ± 0.07	0.44 ± 0.03
	**□**	*P_etef_mttA*_K14	54.4 ± 0.2	0.46 ± 0.00	0.82 ± 0.01	0.57 ± 0.00

^a^ Overall itaconate production rate ([glucose] > 5.5 g L^−1^). ^b^ Maximum itaconate production rate. ^c^ Yield itaconate per consumed glucose.

**Table 4:** Determination of *P_etef_mttA* copy number in five *U. maydis* MB215 ∆*cyp3* ∆MEL ∆UA ∆*dgat* ∆*P_ria1_*::*P_etef_* ∆*fuz7 P_etef_mttA* transformants by quantitative polymerase chain reaction (PCR). ±values indicate the standard error of the mean (*n* = 3).

*U. maydis* Strain	C_t_ Value UMAG_ 02595	C_t_ Value UMAG_ 03726	C_t_ Value *mttA*	Ratio *mttA* to UMAG_ 02595	Ratio *mttA* to UMAG_ 03726	Rounded Mean
wildtype	27.6 ± 0.10	27.9 ± 0.29	35.9 ± 1.20	0.0	0.0	0
∆UMAG_05079::*P_etef_mttA*	25.7 ± 0.14	25.8 ± 0.24	25.7 ± 0.32	1.0	1.0	1
*P_etef_mttA*_K3	28.3 ± 0.12	28.4 ± 0.31	26.7 ± 0.36	3.2	3.1	3
*P_etef_mttA*_K8	27.8 ± 0.10	27.9 ± 0.11	28.5 ± 0.21	0.7	0.6	1
*P_etef_mttA*_K9	28.2 ± 0.27	28.5 ± 0.16	26.4 ± 0.18	3.7	3.9	4
*P_etef_mttA*_K10	26.8 ± 0.17	26.6 ± 0.35	26.6 ± 0.10	1.2	0.9	1
*P_etef_mttA*_K14	26.6 ± 0.03	27.0 ± 0.13	25.2 ± 0.13	2.8	3.3	3

## Data Availability

Data is contained within the article or supplementary material.

## Supplementary Materials

The following are available online at https://www.mdpi.com/2309-608X/7/1/20/s1, Figure S1: Low-density pulsed fed-batch fermentation of *U. maydis* strain K14, Table S1: Oligonucleotides used for deletion and overexpression constructs, Table S2: Production parameters of two engineered *U. maydis* MB215 strains in two different types of fed-batch fermentations.
