# Supplementary material for: An Optimized Ustilago maydis for Itaconic Acid Production at Maximal Theoretical Yield
Source: J Fungi (Basel). 2020 Dec 31;7(1):20. doi: 10.3390/jof7010020 (PMC7824378; doi:10.3390/jof7010020)
Supplement: Supplementary file 1 [file jof-07-00020-s001.pdf]

Journal of *Fungi*

Supplementary materials to:

An Optimized *Ustilago Maydis* for Itaconic Acid Production at Maximal Theoretical Yield

J. Becker[1, §], H. Hosseinpour Tehrani[1, §], P. Ernst[2, §], L. M. Blank[1] and N. Wierckx[2, *]

[1] *iAMB* – Institute of Applied Microbiology, ABBt – Aachen Biology and Biotechnology, RWTH Aachen University, Worringerweg 1, 52074, Aachen, Germany; johanna.becker@rwth-aachen.de; hamed.tehrani@rwth-aachen.de; lars.blank@rwth-aachen.de
[2] Institute of Bio- and Geosciences IBG-1: Biotechnology, Forschungszentrum Jülich, 52425, Jülich, Germany; p.ernst@fz-juelich.de; n.wierckx@fz-juelich.de
* Correspondence: n.wierckx@fz-juelich.de
§ These authors contributed equally to this manuscript

Table S1. Oligonucleotides used for deletion and overexpression constructs.

primer name	sequence (5′-3′) & description
JB-89	ctcgagtttttcagcaagatCCGATCGCTGTTAGGACAC<br>Amplification of 5′-UTR flank for generation of *fuz7* deletion construct
JB-90	acttctggccCGTGAAACGTTGCAAAACAG<br>Amplification of 5′-UTR flank for generation of *fuz7* deletion construct
JB-91	acgtttcacgGGCCAGAAGTTCCTATTC<br>Amplification of FRT_m1-HygR-FRT_m1 cassette for generation of *fuz7* deletion construct
JB-92	tctcagtcggCCCGGGAAGTTCCTATAC<br>Amplification of FRT_m1-HygR-FRT_m1 cassette for generation of *fuz7* deletion construct
JB-93	acttcccgggCCGACTGAGAGATTATGGTC<br>Amplification of 3′-UTR flank for generation of *fuz7* deletion construct
JB-94	aggagatcttctagaaagatAATCGGAACCGTGTACCTG<br>Amplification of 3′-UTR flank for generation of *fuz7* deletion construct
JB-126_fwd	ATGGCTTCTCAATCGCAC<br>Amplification of reference gene UMAG_02592 during qRT-PCR
JB-127_rev	CCTGGTGTTGAGGATGAG<br>Amplification of reference gene UMAG_02592 during qRT-PCR
JB-128_fwd	ACATCGTCAAGGCTATCG<br>Amplification of reference gene UMAG_03726 during qRT-PCR
JB-129_rev	AAAGAACACCGGACTTGG<br>Amplification of reference gene UMAG_03726 during qRT-PCR
JB-132_fwd	AACACGTTCAACTGCGTCAA<br>Amplification of *mttA* during qRT-PCR
JB-133_rev	GAACATGATGGCCGAGGTG<br>Amplification of *mttA* during qRT-PCR
HT-4a_rev	ACAGACGTCGCGGTGAGTTC<br>Verification of FRT-HygR-cassette based insertions
HT-202_fwd	TCCTGCGTCAGTCGTCCAAC<br>Verification of P_{etef}*mttA* integration
HT-203_fwd	GTCCGAGGGCAAAGGAATAG<br>Verification of *fuz7* deletion
HT-210_fwd	TCGCTGTTAGGACACAACTG<br>Amplification of *fuz7* deletion construct
HT-210a	TCGGTGTGCGGCGATTTCTG

	Verification of *fuz7* deletion
HT-211	CCGTGTACCTGGCTGTGTAG<br>Amplification of *fuz7* deletion construct
HT-220	GATTCTGTGGGACAAGAAGC<br>Verification of *fuz7* deletion
Tnos	CAAGACCGGCAACAGGATTC<br>Verification of P_{etef}*mttA* integration

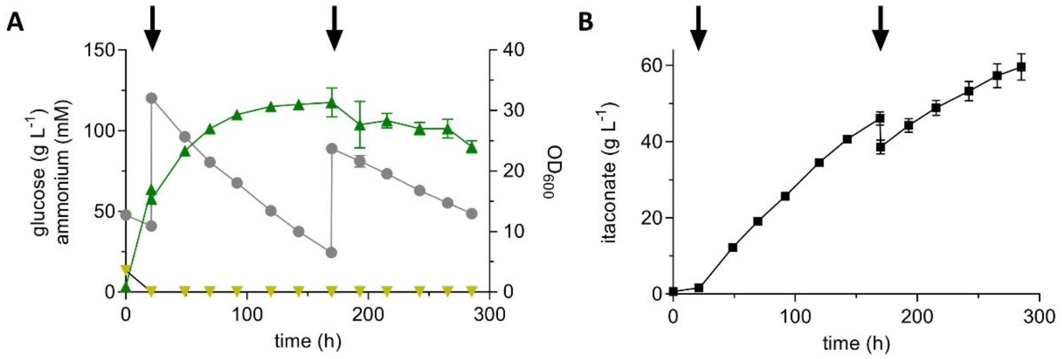

Figure S1. Low-density pulsed fed-batch fermentation of *U. maydis* strain K14. (A) concentration of glucose (●), ammonium (▼) and OD_{600} (▲) and (B) concentration of itaconate (■) during fermentation in a bioreactor containing batch medium with 50 g L^{-1} glucose and 0.8 g L^{-1} NH_4Cl. The pH was kept at 6.5 by automatic titration with NaOH. Arrows indicate the addition of 80 g glucose. Error bars indicate the standard error of the mean (n = 3).

Table S2. Production parameters of two engineered *U. maydis* MB215 strains in two different types of fed-batch fermentations. ±values indicate the standard error of the mean (n = 3 for 50 g L^{-1} glucose fermentation) and the deviation from the mean (n = 2 for 200 g L^{-1} glucose fermentation).

Fermentation conditions	Feed	Strain	ITA titer$_{max}$[a] (g L^{-1})	q_P[b] (g L^{-1} h^{-1})	$y_{P/S}$[c] (g_{ITA} g_{glu}^{-1})
200 g L^{-1} glucose<br>4 g L^{-1} NH_4Cl<br>$CaCO_3$	Pulsed	*U. maydis* MB215 Δ*cyp3* Δ*fuz7* ΔP_{ria1}::P_{etef} P_{etef}*mttA*	220.3	0.46	0.33
	Pulsed	*U. maydis* strain K14	205.6 ± 1.1	0.43 ± 0.00	0.32 ± 0.00
50 g L^{-1} glucose<br>0.8 g L^{-1} NH_4Cl<br>NaOH	Pulsed	*U. maydis* MB215 Δ*cyp3* Δ*fuz7* ΔP_{ria1}::P_{etef} P_{etef}*mttA*	35.9 ± 1.5	0.12 ± 0.00	0.20 ± 0.01
	Pulsed	*U. maydis* strain K14	59.6 ± 5.9	0.21 ± 0.02	0.42 ± 0.02
	Continuous	*U. maydis* strain K14	75.7 ± 1.3	0.24 ± 0.01	0.66 ± 0.02

a. Maximum itaconate titer (g L^{-1}).
b. Overall itaconate production rate ([glucose] > 5.5 g L^{-1}).
c. Yield itaconate per consumed glucose.
